# Draft Genome Sequences of Three *Aeromonas hydrophila* Isolates from Catfish and Tilapia

**DOI:** 10.1128/genomeA.01509-16

**Published:** 2017-01-19

**Authors:** Hasan C. Tekedar, Salih Kumru, Safak Kalindamar, Attila Karsi, Geoffrey C. Waldbieser, Tad Sonstegard, Steven G. Schroeder, Mark R. Liles, Matt J. Griffin, Mark L. Lawrence

**Affiliations:** a College of Veterinary Medicine, Mississippi State University, Mississippi State, Mississippi, USA; b Warmwater Aquaculture Research Unit, Agricultural Research Service, U.S. Department of Agriculture, Stoneville, Mississippi, USA; c Bovine Functional Genomics Laboratory, Agricultural Research Service, U.S. Department of Agriculture, Beltsville, Maryland, USA; d Department of Biological Sciences, Auburn University, Auburn, Alabama, USA

## Abstract

*Aeromonas hydrophila* is a Gram-negative bacterium that is particularly adapted to freshwater environments and can cause severe infections in fish and humans. Here, we report the draft genomes of three *A. hydrophila* catfish and tilapia isolates.

## GENOME ANNOUNCEMENT

*Aeromonas hydrophila* is a Gram-negative opportunistic pathogen that is affiliated with the *Gammaproteobacteria* class in the family *Aeromonadales*. *A. hydrophila* is ubiquitously found in aquatic environments and is an etiologic agent of motile aeromonad septicemia (MAS) in fish. It is also able to cause severe infections in mammals, including humans. *Aeromonas* is a highly diverse genus, inhabiting a variety of aquatic ecosystems and host organisms (1). Since 2009, *A. hydrophila* has been negatively affecting the catfish aquaculture industry in the southeast United States, and our research group has been investigating its molecular mechanisms of disease.

The *A. hydrophila* outbreaks on catfish farms are caused by a clonal group of virulent *A. hydrophila* (vAh) isolates. We have released one complete genome (strain ML09-119) (2) and four draft genomes (strains AL10-121, AL09-79, ML09-121, and ML09-122) that represent this clonal group of vAh affecting catfish (3). For comparison, we have also released one complete genome (strain AL06-06; goldfish isolate) (4) and one draft genome (*A. hydrophila* TN97-08; bluegill isolate) isolated from other fish species (5). Here, we report the draft genomes of three additional *A. hydrophila* genomes for comparison purposes, one isolated from diseased catfish (strain Arkansas 2010) and two isolated from diseased tilapia (strains AL97-91 and MN98-04). Comparative genomics of these strains will enable a better understanding of the variation in virulence genes and antigenic structures of fish-pathogenic *A. hydrophila*.

*A. hydrophila* Arkansas 2010, AL97-91, and MN98-04 were sequenced using an Illumina Genome Analyzer IIx (11,143,909 reads with 301× coverage, 6,018,377 reads with 168× coverage, and 7,578,657 reads with 211× coverage, respectively). Read trimming, error correction, and contig creation were conducted using CLC Genomics Workbench version 6.5.1 (CLC Bio) and version Sequencher 5.4.5 (Gene Codes Corporation). Plasmids were sequenced by the Massachusetts General Center for Computational and Integrative Biology (https://dnacore.mgh.harvard.edu).

The draft genomes and their plasmids were submitted to the NCBI Prokaryotic Genome Annotation Pipeline (PGAP) (6) for annotation. For additional annotation and analyses, draft genomes were submitted to the Rapid Annotations using Subsystems Technology (RAST) server (7, 8). General features of the draft *A. hydrophila* genomes are summarized in Table 1. The average nucleotide identity (ANI mean) against the genomes of previously sequenced strains ML09-119 and AL06-06 was calculated using EDGAR (9). Interestingly, the ANI mean between the genomes of strain Arkansas 2010 and vAh strain ML09-119 indicates that Arkansas 2010 is a member of the vAh clonal group. Strain Arkansas 2010 resulted from vAh-infected fish being transported from Alabama to Arkansas.

**TABLE 1:** Summary of genome sequencing results in the present study

Strain	Genome size (bp)	No. of contigs	Plasmid availability/size	Predicted genes/protein coding sequences	No. of tRNAs	Accession no.	ANI^a^ comparison against *A. hydrophila* ML09-119	ANI comparison against *A. hydrophila* AL06-06
Arkansas 2010	4,973,555	12	No	4,470/4,315	93	LYZH00000000	99.99	96.98
AL97-91	4,830,274	42	Yes/6,741 bp	4,423/4,257	97	LYZF00000000	96.95	97.18
MN98-04	4,882,939	46	Yes/2,867 bp	4,476/4,308	99	LYZG00000000	96.94	97.18

^a^ ANI, average nucleotide identity.

Compared to previously published *A. hydrophila* genomes (2, 4), our findings showed that the genomes of strains Arkansas 2010, AL97-91, and MN98-04 carry toxin-antitoxin replicon stabilization system components. Additionally, the AL97-91 and MN98-04 genomes reveal the uniqueness of these strains, in that they are capable of utilizing taurine.

### Accession number(s).

The draft genome sequences of *A. hydrophila* strains Arkansas 2010, AL97-91, and MN98-04 were deposited in GenBank, and their accession numbers are found in Table 1.
